# Magnetic Resonance-Guided Focused Ultrasound Surgery for Obsessive-Compulsive Disorders: Potential for use as a Novel Ablative Surgical Technique

**DOI:** 10.3389/fpsyt.2021.640832

**Published:** 2021-04-06

**Authors:** Kyung Won Chang, Hyun Ho Jung, Jin Woo Chang

**Affiliations:** Department of Neurosurgery & Brain Research Institute, Yonsei University College of Medicine, Seoul, South Korea

**Keywords:** deep brain stimulation, gamma knife surgery, magnetic resonance-guided focused ultrasound, obsessive-compulsive disorder, psychosurgery

## Abstract

Surgical treatment for psychiatric disorders, such as obsessive-compulsive disorder (OCD) and depression, using ablative techniques, such as cingulotomy and capsulotomy, have historically been controversial for a number of scientific, social, and ethical reasons. Recently, with the elucidation of anatomical and neurochemical substrates of brain function in healthy controls and patients with such disorders using various functional neuroimaging techniques, these criticisms are becoming less valid. Furthermore, by using new techniques, such as deep brain stimulation (DBS), and identifying more precise targets, beneficial effects and the lack of serious complications have been demonstrated in patients with psychiatric disorders. However, DBS also has many disadvantages. Currently, magnetic resonance-guided focused ultrasound surgery (MRgFUS) is used as a minimal-invasive surgical method for generating precisely placed focal thermal lesions in the brain. Here, we review surgical techniques and their potential complications, along with anterior limb of the internal capsule (ALIC) capsulotomy by radiofrequency lesioning and gamma knife radiosurgery, for the treatment of OCD and depression. We also discuss the limitations and technical issues related to ALIC capsulotomy with MRgFUS for medically refractory OCD and depression. Through this review we hope MRgFUS could be considered as a new treatment choice for refractory OCD.

## Introduction

Obsessive-compulsive disorder (OCD) is a mental disorder characterized by time-consuming, distressing obsessions and/or compulsions (1). Obsessions are repetitive and persistent thoughts, images, impulses, or urges that are intrusive and unwanted and are commonly associated with anxiety (1). Compulsions are repetitive behaviors or mental acts that the individual feels driven to perform, according to strict rules, in response to an obsession or to achieve a sense of “completeness.” It is often accompanied by avoidance behavior (1).

Most patients experience a continuous symptom course, although there is a waxing and waning symptom pattern in up to 25% (2). However, symptom progression can be highly influenced by treatment. Individuals with OCD often have a poor quality of life, similar to individuals with schizophrenia (3). The burden of personal and socio-economic costs associated with OCD is also considerable (4). Nevertheless, the condition is often unrecognized, particularly because symptoms are often internally experienced rather than externally expressed, and patients may be unwilling to reveal thoughts or behaviors that they perceive as shameful or embarrassing (5). Consequently, the mean time from OCD symptom onset to initial pharmacological treatment is nearly 8 years (6). Among patients who receive clinical attention, <40% receive OCD-specific therapy and <10% receive evidence-based treatment (7).

The initial treatment for OCD involves pharmacological treatment or cognitive-behavioral therapy (CBT) (1, 5, 8). Selective serotonin-reuptake inhibitors (SSRIs) are the first-line of pharmacological treatment for OCD, with proven efficacy in reducing OCD symptoms and tolerability demonstrated in multiple trials (9). However, approximately half of OCD patients treated with first-line treatment fail to show a complete response (10). Insufficient response after CBT or SSRI monotherapy can also be resolved by combination therapy (5, 8). For treatment-refractory OCD, after failure of SSRI or combination therapy, alternative approaches could be considered. Neurosurgical techniques, focused on the lesioning of specific components of the neural circuits associated with OCD, have been used for decades to treat patients with severe, treatment-refractory symptoms (11–13). In this reviews, we provide an overview of the history of surgery for psychiatric disorders and discuss the latest surgical options for psychiatric disorders, with a focus on OCD. To our knowledge there are few review regarding MRgFUS as a treatment option for psychiatric disorders.

## Brief History of Psychiatric Neurosurgery

Surgery for psychiatric disorders includes techniques involving neuroablation and neuromodulation, such as stimulation techniques. Psychiatric neurosurgery has a long history of controversy for various scientific, social, and ethical reasons. Throughout the 1800s, new knowledge regarding functional neuroanatomy and neurophysiology sparked an interest in psychiatric neurosurgery (14). In 1819, Franz Joseph Gall published his thesis on phrenology, suggesting that the brain has distinct functional areas (15). Although the concept of phrenology was defective and was eventually discounted, the idea of neurological functions having an anatomical correlation was expanded on by the seminal work on the localization of language function by Paul Broca and Carl Wernicke, followed by further research by Gustav Fritsch, Eduard Hitzig, and David Ferrier on the localization of the motor cortex (16). These findings inspired interest in psychiatric neurosurgery. In 1889, Gottlieb Burckhardt presented his operative findings and outcomes at the Berlin Medical Congress (17). In six patients with various psychiatric disorders, he conducted selective removal of the left frontotemporal cerebral cortex. In three of these cases, Burckhardt claimed to achieve success in treatment, but his eccentric research was deeply criticized by the international medical community when he published his surgical results in 1891, leading to discontinuation of the project. Research on and practice of psychiatric neurosurgery stagnated until 1935, when a primate model study by John Fulton and Carlyle Jacobsen described the frontal lobe's role in short term memory, anxiety, and aggression (14). This study encouraged Egas Moniz and Almeida Lima to perform the first prefrontal leucotomies in 20 psychiatric patients with schizophrenia, bipolar disorder, and anxiety disorders (18). Walter Freeman and James Watts modified the technique and began to perform modified lobotomies in 1936 (14, 17, 19), which became popular worldwide. By 1949, an estimated 10,000 lobotomies had been performed in the US and Europe. Moniz won the 1949 Nobel Prize for medicine for the discovery of the role of prefrontal leucotomy in the treatment of certain psychoses (17).

Nevertheless, eventually both professional and public opinion turned against lobotomy, as the procedure was regarded as unethical and unscientific. Additionally, the associated morbidity and mortality, the so called “post-leucotomy syndrome,” became more evident (14, 20). To reduce the morbidity and mortality, a more selective approach was considered, and improvements were attained with advances in stereotactic neurosurgery. In 1949, Talairach announced the use of a stereotactic frame to coagulate the frontothalamic fibers in the ALIC selectively, at the 4th International Congress of Neurology in Paris (17). Thereafter, stereotactic psychiatric neurosurgery rapidly replaced prefrontal lobotomy and was applied for various psychiatric disorders (21): cingulotomy for addiction, bipolar disorder, depression, OCD, schizoaffective disorder, and schizophrenia; anterior capsulotomy for general anxiety disorder and OCD; subcaudate tractotomy for depression, OCD, and schizophrenia; anterior callosotomy for schizoaffective disorder and schizophrenia; amygdalotomy for aggressive behavior associated with mental impairment; thalamotomy for Tourette syndrome and hypothalamotomy for addiction, aggression, and sexual disorders.

In the 1950s and 1960s, the use of psychiatric neurosurgery declined, and the number of patients requiring stereotactic psychiatric neurosurgery was markedly reduced (22, 23). This can be ascribed to multiple factors, such as social attitudes and ethical problems, as well as the introduction of psychoactive drugs, such as chlorpromazine, reserpine, lithium, haloperidol, imipramine, and diazepam. The development and approval of other antipsychotics and antidepressants soon followed, and there was evidence that medical therapy was more effective, safer, and cheaper than psychiatric neurosurgery (14).

Although there was controversy surrounding the evolution of psychiatric neurosurgery, it was instrumental in the development of modern standards of research and ethics, with the passing of the National Research Act of 1974 and subsequent publication of the Belmont Report (14). The 1977 US National Commission Report on Psychiatric Neurosurgery claims “*The Commission affirms that the use of psychosurgery for any purpose other than to provide treatment to individual patients would be inappropriate and should be prohibited. Accordingly, the Commission is recommending safeguards that should prevent the performance of psychosurgery for purposes of social or institutional control or other such misuse.”* *(**24**)*.

## Various Neuroablative Procedures for Obsessive-Compulsive Disorder

Various neuroablative surgeries have been performed for OCD. Each surgery is named for its target, such as capsulotomy targeting the internal capsule, cingulotomy targeting the anterior cingulate cortex, and subcaudate tractotomy targeting the subcaudate white matter. Limbic leucotomy involves a combination of the latter two procedures (5, 12, 21). The concept underlying ablative surgery for OCD is based on the pathological hyperactivity and dysfunctional connectivity in the limbic cortico-striato-thalamo-cortical (CSTC) loop (25).

Currently, the ablative methods used most commonly are radiofrequency and radiosurgical ablation, as well as the novel magnetic resonance-guided focused ultrasound surgery (MRgFUS) (21, 26–30). Approximately 30–60% of patients with intractable OCD may experience significantly reduced OCD symptoms post-operatively (1). However, only gamma ventral capsulotomy has been studied in a double-blind, sham-controlled randomized trial (31). Using the response criteria [a 35% reduction in the baseline Yale–Brown Obsessive Compulsive Scale (Y-BOCS) score plus a Clinical Global Impression change score of 1 or 2], the primary outcome measure did not reach statistical significance after 12 months, although the Y-BOCS score reduction over that same follow-up period was significantly greater in the active treatment group. At the end of the follow-up period (54 months), 7 of 12 (58%) patients who underwent radiosurgery were deemed responders.

### Cingulotomy

The anterior cingulate cortex is a region in the medial prefrontal cortex that surrounds the rostral corpus callosum. This region seems to have emotional and cognitive functions. Both functional roles make the anterior cingulate cortex a theoretically ideal focus for therapeutic intervention in psychiatric disorders in which these processes are impaired (25).

After Egas Moniz announced his first prefrontal leucotomy in 1936, thousands of patients with mental disorders underwent frontal lobar surgery until the 1950s (32), with modifications introduced by Walter Freeman and James Watts (19). However, because of the lack of anatomical precision of frontal leucotomy and the emergence of side effects, more restricted surgeries, such as frontal topectomy or undercutting under direct visualization or by electrocoagulation, were developed (33). After the stimulation of Brodmann area 24 in monkeys showed autonomic changes in emotional expression and a widespread cortical suppressor effect (34), bilateral removal of the anterior part of the cingular gyrus, i.e., anterior cingulotomy, was performed by Whitty et al. (35). Among the 24 patients in their series, four OCD patients showed significant improvements. Subsequently, in 1962, Foltz and White reported that stereotaxic cingulotomy in patients with intractable pain also improved their anxiety or depression (36). With these impressive results, Ballantine et al. performed bilateral stereotaxic cingulotomy for 57 patients with mental disorders (32) in 1967, and showed variable improvements in 30 patients. In 1972, Laitinen et al. used a stereotaxic frame for cingulotomy (37), and in 1990, radiofrequency was first used by Hassenbusch et al. (38). According to Ballantine et al.'s large study in 1987 (39), more than 400 OCD patients underwent bilateral stereotactic cingulotomy. The reported overall success rate was 30–40%, with no other major side effects, but some minor complications were identified, such as persistent headache, nightmares, decreased libido, weight gain, and urinary incontinence. Adverse effects after cingulotomy have been reported as transient to mild symptoms, such as urinary symptoms (14%), as well as permanent to serious symptoms, such as epilepsy or cognitive changes (5.2%) (40).

In our institute's series regarding cingulotomy for OCD (28, 41), 17 patients had a Y-BOCS score of 35.0 ± 3.86 preoperatively and showed a mean improvement of 48% over the baseline score after a follow-up period of at least 2 years. A neuropsychological examination was performed in all of our patients to investigate whether there were any differences in cognitive function at 24 months after cingulotomy. No significant adverse effects were observed after the surgery. In a recent long-term study with a 5-year mean follow-up period in 64 patients, Sheth et al. (42) showed a 47% rate of full response (more than 35% improvement in the Y-BOCS score).

### Capsulotomy

Disruption of the ALIC is thought to yield efficacious or therapeutic relief in some psychiatric disorders, and some studies have demonstrated orbitofrontal cortex and subgenual cingulate projections leading to the medial thalamic nucleus. In 1949, Talairach first introduced the technique of anterior capsulotomy to disconnect fibers from the orbitofrontal cortex leading to the limbic system that pass through the ALIC for patients with psychiatric disorders (17). In the 1950s, Leksell and Talairach modernized anterior capsulotomy (43). In 1953, the first radiosurgical capsulotomy was performed with 300-kV X-rays (44). Lippitz et al. reported 22 cases of bilateral thermo-capsulotomy from 1976 to 1989, with a median follow-up period of 8.4 years; beneficial results were seen in 47% of cases (45). Bilateral thermo-capsulotomy results from a recent large study were reported by numerous studies. The rate of improvement in Y-BOCS scores was 43.3% according to Oliver et al. (*n* = 10) (46), 80.9% according to Liu et al. (*n* = 35) (47), and 31.4% according to D'Astous et al. (*n* = 19) (48). The adverse effects after capsulotomy were reported as transient to mild in 56.2% of patients and as permanent to serious in 21.4% of patients in another study (40). Leksell introduced capsulotomy via gamma knife radiosurgery in 1955 (44). Improvements after gamma knife anterior capsulotomy were observed in 55–70% of patients. However, anatomical target localization, dose, and collimator selection varied among reports (44). Rück et al. in their series, compared thermo-capsulotomy and gamma capsulotomy (49) and found some side effects, such as apathy, incontinence, seizure, and executive dysfunction; they also reported that a very high radiation dose or multiple procedures should be avoided. Additionally, larger targets using an 8-mm collimator showed adverse radiation effects (50). Gamma capsulotomy has a risk of adverse radiation effects, such as radiation necrosis or brain edema or cyst formation (49). Worldwide, more than 240 cases of gamma capsulotomy were performed, and if the maximal dose was reduced below 180 Gy, the number of adverse effects decreased (51). In a recent double-blind, randomized controlled trial by Lopes et al., 58.3% (7/12) of cases were deemed responders after gamma ventral capsulotomy (31). Recently, in a large study of capsulotomy by Rasmussen et al. (52) 31 out of 55 patients had an improvement in the Y-BOCS score in a primary measure, and over 35% improvement in 3-year follow-up period. Patients had significant improvements in depression, anxiety, quality of life, and global functioning without significant acute adverse effects, only 5% patients developed radio-necrotic cysts in long term follow-up. As the capsulotomy procedure have advanced, the adverse events have decreased significantly, with a remaining favorable treatment response of ~50 ~ 66% of patients which were treated at experienced centers (53). The reduction in the severity of OCD may also result from improved efficacy of pharmacological and psychological treatments that work synergistically with capsulotomy as well as direct modulation of the OCD neural pathways (53).

### Limbic Leukotomy

Limbic leucotomy is a combination of anterior cingulotomy and capsulotomy or subcaudate tractotomy. Kelly et al. (54) performed limbic leucotomies starting in the 1970s, with proven efficacy for mood disorders and OCD. Depending on the clinical symptoms, limbic leucotomy can be performed as a one-stage procedure (55), in which frontothalamic white matter tracts of the basal medial frontal lobes are lesioned, along with lesioning in anterior cingulate cortex. Alternatively, it can be performed in a two-stage procedure involving initial anterior cingulotomy, followed by subcaudate tractotomy (56). In a 7-year prospective study of patients with intractable bipolar disorder, one-stage limbic leucotomy yielded a significant reduction in depressive symptoms but not in manic symptoms (57). One group who performed a two-stage procedure showed a 73% symptom improvement rate in patients with OCD and intractable major depressive disorder who did not initially respond to anterior cingulotomy alone (56). The side effects of limbic leucotomy appear to be transient and to resolve spontaneously; they include transient hallucinations, amnesia, and mania (42). In particular, however, abulia appears to develop at a higher rate following limbic leucotomy than following cingulotomy, but it is still typically self-limiting (57).

## Neuromodulative Surgery—Deep Brain Stimulation for Obsessive-Compulsive Disorder

DBS is an adjustable, reversible, and non-destructive procedure that has been proven to be safe for treating movement disorders. With DBS, surgically implanted electrodes deliver controlled electrical pulses to targeted areas of the brain, which activate adjacent neural circuits (58). The settings of the stimulation can be changed, and the electrodes can be removed from the brain. Compared to ablative neurosurgery, DBS has the advantages of being reversible and adjustable. Thus, there has been a trend for replacement of ablation techniques by DBS.

Significant advances in neuroscience and neuroimaging have resulted in the discovery of some circuits in the brain (59), and the application of DBS has increased with the greater understanding of the pathophysiology of psychiatric disorders.

DBS is reserved for highly refractory OCD cases, i.e., < ~1% of patients (60). Approximately 30–50% of patients with severe refractory OCD respond to these alternative treatments (13, 61).

DBS has been attempted using various targets. Most studies on DBS have targeted striatal areas, including the ALIC, ventral capsule (VC), ventral striatum (VS), nucleus accumbens or the ventral caudate nucleus, subthalamic nucleus, and inferior thalamic peduncle (1, 13, 62, 63). Preliminary studies have also described the potential benefit of non-striatal targets, such as the superolateral branch of the medial forebrain bundle (64).

DBS was initially applied in the ALIC by Nuttin et al. (65), and reported DBS results for OCD: the mean preoperative Y-BOCS score of 32.3 ± 3.9 decreased to 19.8 ± 8.0 post-operatively, and the stimulation effect was maintained for at least 21 months after DBS. The effect of DBS in the internal capsule was limited and required high power, resulting in high battery consumption. Thus, the DBS target was gradually shifted more posteriorly where the anatomical sites were close to the VS, VC, nucleus accumbens, inferior thalamic peduncle, and bed nucleus of the stria terminalis (66, 67). Greenberg et al. (68) targeted the VC/VS where more compact fibers of the CSTC circuit course, slightly posterior to the target of standard capsulotomy. Additionally, the effects of DBS were more pronounced with posterior stimulation. The more posteriorly the electrode was positioned, the less energy (voltage and pulse width) was needed.

The nucleus accumbens plays a major role in modulating the CSTC circuit. Sturm et al. (62) designed a double-blind sham-controlled crossover study with unilateral DBS for the nucleus accumbens. Five of 10 patients showed reduction of more than 25% over the baseline Y-BOCS score, and one patient showed a reduction of > 35% at 1 year post-operation. Another study on bilateral nucleus accumbens DBS in 16 patients by Denys et al. (69) reported 47 and 52% reductions from the baseline Y-BOCS score after 12 and 21 months, respectively. Bilateral DBS in the inferior thalamic peduncle and in the limbic part of the subthalamic nucleus has also been reported, but the patients included were somewhat diverse and the sample size was small (67, 70).

Surgical risks still exist, and both lesioning and DBS pose a risk of hemorrhage, seizures, and infection. Although the risk of intracerebral hemorrhage is inevitable, the occurrence rates are low (1–2% in large studies), irrespective of the symptoms (71). Additionally, the implantable pulse generator should be replaced when the battery is depleted.

## New Emerging Techniques—Magnetic Resonance-guided Focused Ultrasound Surgery for Obsessive-Compulsive Disorder

Several experimental studies on focused ultrasound lesioning were conducted in the 1950s (72, 73). Owing to skull anatomy and the characteristics of ultrasound, focused ultrasound was only applied during craniotomy until the 1990s. The skull acts as a barrier and is a major obstacle against the penetration of ultrasound for making a focal lesion in the brain, and the heat from energy absorption can damage the scalp, bone, and adjacent brain parenchyma (74). The development of the phased array transducer and the introduction of MR thermometry made it possible to use transcranial MRgFUS as an ablative method (75). Unlike stereotactic radiofrequency procedures, which are irreversible, MRgFUS lesioning allows modification during the procedure since the thermal area can be monitored in real-time.

Transcranial MRgFUS is a minimally invasive and real-time monitoring procedure that has advantages over surgical techniques, such as radiosurgery, and other lesioning procedures (26). Intermittently examining the MR image or using MR thermometry during the sonication can confirm or predict the lesioning, and no ionizing radiation is involved. In addition, its precision and the feasibility of real-time monitoring during the procedure can minimize procedure-related complications (76). In addition, there are no other concerns about hardware implantation or replacement, or surgical morbidity, as with DBS. MRgFUS is a potentially more viable and cost-effective alternative to radiofrequency capsulotomy (26). Recently, MRgFUS has been widely applied for the treatment of various movement disorders (essential tremors, etc.), but there have been few studies involving psychiatric disorders. In our institute, MRgFUS for OCD has been done since our first study in 2013 (27).

### Patient Selection and Inclusion Criteria

Patients who were diagnosed as treatment-refractory OCD were selected. All patients failed to respond to more than three SSRIs, including clomipramine, and more than one antipsychotic drug augmentation strategy (one or more SSRIs and one or more antipsychotics) and had OCD symptoms with psychosocial impairment for at least 5-years. Experienced psychiatrists conducted psychiatric interviews for diagnoses and assessment of symptoms or psychosocial functions. The inclusion criteria of the patients are: (1) a primary OCD diagnosis according to the Structured Clinical Interview of the Diagnostic and Statistical Manual of Mental Disorders; (2) at least a 3-year history of OCD symptoms with psychosocial dysfunction (determined by a Global Assessment of Functioning Scale, score ≤ 50); (3) a minimum score of 28 on the Yale–Brown Obsessive-Compulsive Scale (Y-BOCS); and (4) treatment refractory status, that is, non-responsive to pharmacological treatment (more than two types of serotonin reuptake inhibitors at the maximum tolerated dose for more than 12 weeks) and cognitive-behavioral therapy [a minimum of 20 sessions of primarily therapist-guided Exposure and Response Prevention (ERP)]. Previous medication (SSRIs or antipsychotics) were kept unchanged for at least 30 days before MRgFUS capsulotomy, and the regimen and dosage were maintained until the final assessment from the follow-up visit.

### MRgFUS Capsulotomy: Procedures & Targeting

**Figure 1:**
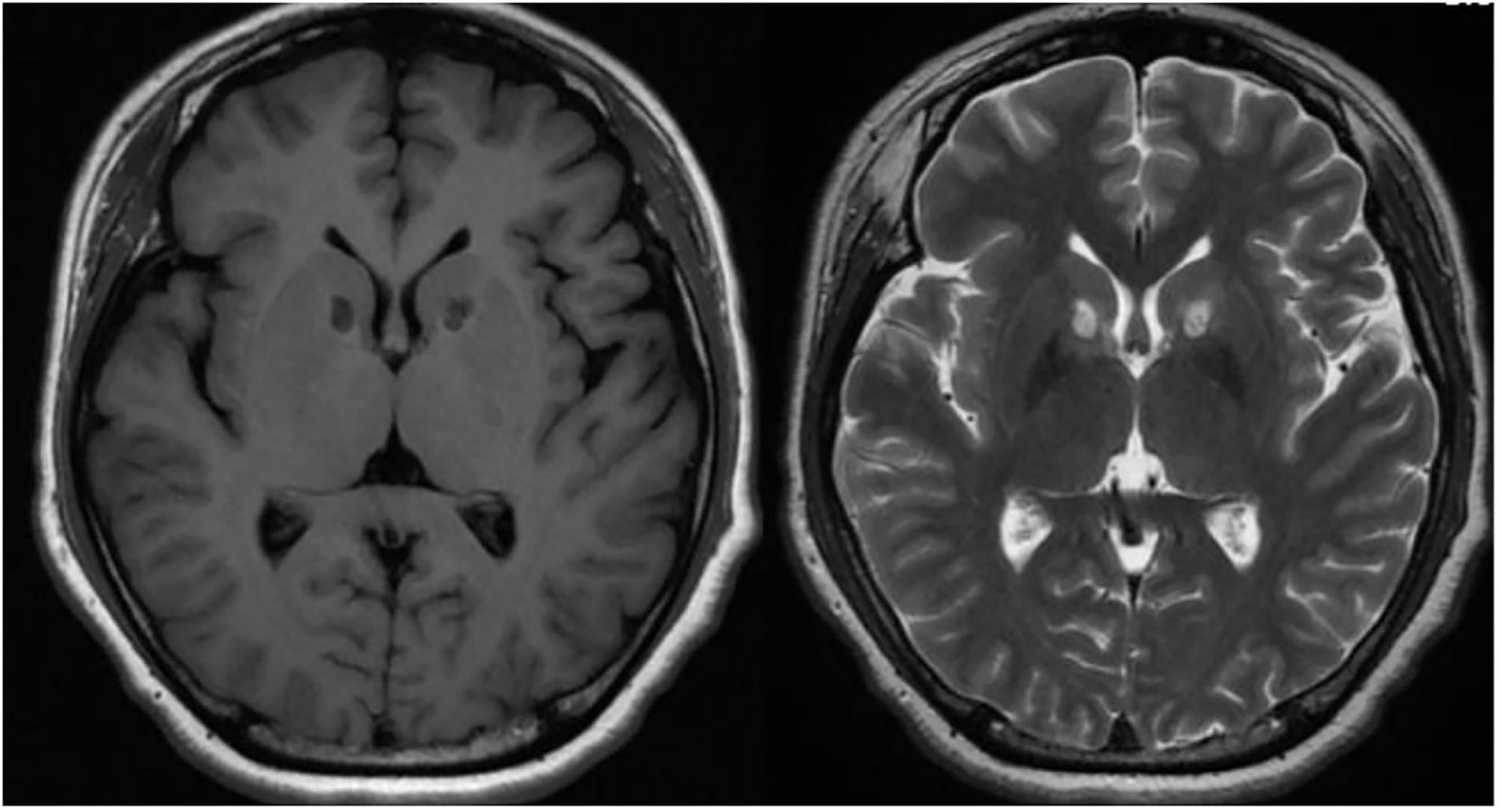
One-week post-operative MR image of a patient with MRgFUS Capsulotomy.

MRgFUS capsulotomy was performed using a 3-T MR system (GE Medical Systems, Milwaukee, WI, USA) and the ExAblate 4,000 system (InSightec, Haifa, Israel) which features a 30-cm diameter, hemispherical, 1,024 element phased-array transducer operating at 650 kHz. The patient's scalp is completely shaved and after the fixation of the Cosman–Roberts–Wells stereotactic frame, the patient is positioned to the ExAblate 4,000 system. A presonication MRI is performed and the images are fused with computed tomography, and other MR sequences are also evaluated to determine the target coordinates. The bilateral ALIC is targeted from the location 7-mm anterior to the anterior margin of the anterior commissure in the level of AC–PC (Anterior Commissure—Posterior Commissure) plane, extending 2–3 mm along the internal capsule in the coronal view. Several subthreshold heatings with low-power sonications of 10 s durations were applied to induce peak temperatures of 40–42°C. This low power sonication procedure is for safety which allows us to visualize the exact position and size of the thermal spot and to review the overall safety profile of the applied sonication parameters. After confirming the exact target, high-power sonication was then applied in a repetitive process guided by MRI and MR thermometry, with gradual increase in acoustic power and energy to achieve a peak temperature of 51–56°C in the target region for more than 3 s. By adjusting the target coordinates, the goal is to create an ~10-mm elliptical lesion in each side of the bilateral ALIC. During the procedure, particularly during cooling time, the patient is asked questions and examined by a neurosurgeon and a psychiatrist to observe whether there are notable physical or psychological changes. The patient is fully awake during the whole procedure and is monitored as an inpatient for 24 h after the procedure (Figure 1).

In study of treatment-refractory OCD in 11 patients who underwent MRgFUS in our institute (30), the Y-BOCS score decreased significantly over the 24-month follow-up period (mean ± standard deviation, 34.4 ± 2.3 at baseline v. 21.3 ± 6.2 at 24 months, *p* < 0.001). In addition, scores on the Hamilton Rating Scales for Depression (HAM-D) and Anxiety (HAM-A) also significantly decreased from baseline over the 24-month period (HAM-D, 19.0 ± 5.3 v. 7.6 ± 5.3, *p* < 0.001; HAM-A, 22.4 ± 5.9 v. 7.9 ± 3.9, *p* < 0.001). The Global Assessment of Functioning (GAF) scores improved significantly (35.8 ± 4.9 at baseline v. 56.0 ± 10.3 at 24 months, *p* < 0.001). Other neuropsychological function test scores, such as Wechsler Adult Intelligence Scale-Korean version scores, Memory Quotient scores, and Digit Span test-forward scores, were significantly improved, while other test scores, such as the Controlled Oral Word Association test scores, Stroop test scores, and Digit Span test-backward scores, remained unchanged. Compared to the pre-sonication condition, high-resolution fluorodeoxyglucose positron emission tomography of the eight patients showed significantly decreased uptake in the orbitofrontal cortex (*p* < 0.001). The side effects of MRgFUS included headache and vestibular symptoms, but these were mild and temporary. Thus, these findings showed that bilateral thermal lesioning of the ALIC using MRgFUS may improve obsessive-compulsive, depressive, and anxiety symptoms in patients with treatment-refractory OCD, without serious side effects.

In our very recent study (77), we successfully performed bilateral thermal capsulotomy with MRgFUS in four patients with treatment-resistant depression (TRD). All patients underwent MRgFUS without serious side effects. During the 12-month follow-up period, all four patients experienced reduced symptoms and improved functional scores: the Beck Depression Inventory and Hamilton Depression Scale (HAM-D) scores decreased by 61.2 and 83.0%, respectively, and the GAF total score improved by more than 50%. Our results were consistent with those of TRD patients who underwent conventional radiofrequency capsulotomy, although we observed faster improvement in our patients. Three of the four patients experienced a faster decrease in symptoms within 1 week of MRgFUS, which may be due to post-procedure anxiety relief. In addition, all participants were able to continue their daily activities from the day after the procedure, without complications. These results suggest the possibility that capsulotomy by MRgFUS can also improve various psychiatric disorders.

In a recent study by Davidson et al. (78). MRgFUS capsulotomy was performed in 12 patients (six patients with OCD and six patients with MDD) without serious clinical or radiographic adverse events. For OCD, a 35% reduction in the Yale-Brown Obsessive-Compulsive Scale and for MDD, a 50% reduction in the 17-item Hamilton Depression Rating Scale was observed.

However, as research is still insufficient, in order not to repeat the mistakes of the previous history of psychiatric neurosurgery, it is important to conduct research on MRgFUS capsulotomy only in institutions with teams of well-experienced functional neurosurgeons and psychiatrists.

To date, the application of MRgFUS for numerous brain targets for treating psychiatric disorders has been limited (29, 76) in patients with a low skull-density ratio (which represents a skull that is difficult to penetrate by ultrasound) and with lateral targets (on which it is difficult to focus ultrasound). Nevertheless, ongoing technical development may eventually overcome these limitations.

## Conclusion

Among the currently available surgical techniques, MRgFUS is emerging as a promising ablative surgical method. Further larger cohort studies are required to compare the surgical methods in future.

## Author Contributions

KC wrote the paper. HJ and JC provided the idea and reviewed the paper. All authors contributed to the article and approved the submitted version.

## Conflict of Interest

The authors declare that the research was conducted in the absence of any commercial or financial relationships that could be construed as a potential conflict of interest.
